# Self-awareness protects working memory in people under chronic stress: An ERP study

**DOI:** 10.3389/fpsyg.2022.1003719

**Published:** 2022-09-30

**Authors:** Wenjuan Xing, Shu Zhang, Zheng Wang, Dan Jiang, Shangfeng Han, Yuejia Luo

**Affiliations:** ^1^College of Economics and Management, Qilu Normal University, Jinan, China; ^2^College of Teacher Education, Qilu Normal University, Jinan, China; ^3^Zhuhai Sanzao Central Primary School, Zhuhai, China; ^4^Department of Psychology and Center for Brain and Cognitive Sciences, School of Education, Guangzhou University, Guangzhou Province, China; ^5^Center for Brain Disorders and Cognitive Sciences, Magnetic Resonance Imaging Center, Shenzhen University, Shenzhen, China; ^6^The State Key Lab of Cognitive and Learning, Faculty of Psychology, Beijing Normal University, Beijing, China; ^7^The Research Center of Brain Science and Visual Cognition, Kunming University of Science and Technology, Kunming, China

**Keywords:** self-awareness, working memory, chronic stress, P2, N2, LPP

## Abstract

Chronic stress impairs working memory (WM), but few studies have explored the protective factors of the impairment. We aimed to investigate the effect of self-awareness on WM processing in people under chronic stress. Participants under chronic stress completed an n-back task after a self-awareness priming paradigm during which electroencephalograms were recorded. The behavioral results showed that participants whose self-awareness was primed reacted faster and more accurately than the controls. Event-related potentials (ERPs) revealed the following (1) P2 was more positive in the self-awareness group than in the controls, indicating that self-awareness enhanced allocation of attention resources at the encoding stage. (2) N2 was attenuated in the self-awareness group compared with the controls, indicating that smaller attention control efforts were required to complete WM tasks adequately after self-awareness priming; and (3) enhanced late positive potential (LPP) was evoked in the self-awareness group compared with the controls, suggesting self-awareness enabled participants to focus attention resources on the information at the maintenance stage. Critically, mediational analyses showed that LPP mediated the relationship between self-awareness and WM response times. This result suggests that the fact that participants whose self-awareness was primed were able to achieve better behavioral performances may be attributed to their mobilization of sustained attention resources at the maintenance stage. In summary, self-awareness exerted a protective effect on WM in those under chronic stress, which may be due to the enhancements in the allocation and mobilization of attention. These results could be used to develop more specific coping strategies for people under chronic stress.

## Introduction

Working memory (WM) is a fundamental process underlying higher cognitive functions, such as learning, language comprehension and problem solving. Thus, WM has an important significance to an individual’s adaptation and survival (Wagner, 1999; Baddeley, 2003). Abundant evidence suggests that exposure to sustained stress degrades WM (Evans and Schamberg, 2009; Marin et al., 2011; Young and Yukiori, 2015). Such an effect is thought to result from poor attention resources allocation during WM processing. Researchers argue that stress results in more difficulty when inhibiting task-irrelevant internal thoughts which negatively affect attention resources, and therefore lead to impaired WM (Bishop, 2008; Schoofs et al., 2008; Liston et al., 2009; Qin et al., 2009). However, few studies inform us how one might protect WM from the harmful effects of stress. Here, we explore whether self-awareness is a protective factor for these harmful effects.

Self-awareness refers to a state in which individuals pay attention to themselves and experience heightened awareness of their own internal feelings and beliefs (Duval and Wicklund, 1972). The current consensus is that self-awareness has important implications for self-evaluation and motivation (Gendolla et al., 2008; Carver and Scheier, 2012; Silvia and Phillips, 2013). It leads people to compare themselves to certain standards and endows success with more necessity and value, thereby mobilizing more resources to meet these standards (Silvia et al., 2010, 2011). Many studies have supported this viewpoint (Alberts et al., 2011; Huang et al., 2017; Xu et al., 2021). One example is the study by Alberts et al. (2011), which found that cognitive resources can be protected by priming self-awareness to circumvent the occurrence of ego depletion. Therefore, we presumed that high self-awareness can also protect attention resources, offsetting the harmful effects of chronic stress on WM. Moreover, data from neuroimaging studies suggest that inducing self-awareness can activate the prefrontal cortex (PFC) region and contribute to regulating attention in this area which is implicated in WM (Lei et al., 2011; Vago and David, 2012).

Although some people are more self-aware than others in trait (this trait is called self-consciousness) (Fenigstein et al., 1975), self-awareness can be temporarily increased by various manipulations, such as seeing oneself in the mirror (Wiekens and Stapel, 2008; Bender et al., 2018) or priming self-related personal pronouns (Walla et al., 2007; Xu et al., 2021). The scrambled sentence task (SST) is an implicit paradigm that has been shown to effectively increase self-awareness. In the SST, participants were asked to unscramble the scrambled sentence that began with the word I (e.g., I buy some bread) in a self-awareness condition. In the other-awareness (i.e., control) condition, participants were asked to unscramble the neutral scrambled sentence that began with no self-related personal pronouns (e.g., He or She buys some bread).

Event-related potentials (ERPs) have been widely used to assess alterations in the dynamic time course of neural activity during WM processing (Picton et al., 1995; Key et al., 2005). Woodman and Vogel (2005) demonstrated that encoding and maintenance operated independently in WM (Woodman and Vogel, 2005). P2 and N2 are ERP components that are associated with the early encoding stage. Specifically, P2 is related to the early allocation of attention and the initial stage of updating (Crowley and Colrain, 2004; Celeste D Lefebvre et al., 2005). Indeed, participants with a lower score in WM tasks have shown smaller P2 (Francisco et al., 2021). N2 is more sensitive to attentional control and is closely associated with the automatic processing of response-inhibition (Folstein and Petten, 2008; Qi et al., 2018; Xu et al., 2022). Enhanced N2 activity has been observed as an index of the difficulty in target and non-target discrimination and is considered highly suggestive of the allocation of “attentional effort” (Crego et al., 2009; Couperus et al., 2021). Altogether, we expected self-awareness to cause dissociable effects on attention resources allocation and attentional control processing at the early encoding stage, resulting in larger P2 and smaller N2 activities. Late positive potential (LPP) is a dynamic measure of the sustained allocation of attentional resources to visual stimuli, with close links to memory encoding and maintenance processing (Ruchkin et al., 1990; Weinberg and Greg, 2011; Ma et al., 2019). Thus, we expected that self-awareness would evoke more positive LPP.

In summary, the purpose of the current study was to explore the influence of priming self-awareness on WM in people under chronic stress. Participants were divided into two groups (self-awareness condition and control condition), and completed the implicit self-awareness priming task (i.e., the SST) or control task, respectively. Subsequently, all participants were asked to complete an n-back task, during which time behavior and electroencephalogram (EEG) were measured (Michael et al., 2007; Aubry et al., 2018). Based on these aforementioned observations and analyses, we expected that self-awareness would enable individuals to be protected from the harmful effects of chronic stress on WM. This protective effect may occur due to the enhancement in attention resources allocation.

## Materials and methods

### Participants

The present study specifically sought to explore the impact of self-awareness on WM in people under chronic stress. The relationship between self-awareness and WM among people with a low stress level will be reported elsewhere. Participants were recruited from a local university through online and public postings. All postings specified the inclusion criteria of the study: (1) graduates who were preparing for the CNPEE (an important and competitive exam in the Chinese educational system) from May 2021 to December 2021 because many previous studies have supported that examination preparation is a long-term stressor (Liston et al., 2009; González-Cabrera et al., 2014; Yuan et al., 2016); (2) physically healthy people; (3) those with normal or corrected-to-normal vision and normal hearing ability; (4) those with no habit of staying up late; (5) those with no history of psychiatric or neurological illness or psychoactive drug use. All eligible participants were further screened by the following exclusion criteria: completed the Perceived Stress Scale (PSS) and Student-Life Stress Inventory (SLSI), ensuring that all participants were exposed to chronic stress (PSS top 25%) and not exposed to any other major stressors. Participants were first screened based on the inclusion criteria at the time of recruitment and were further screened by the exclusion criteria by a self-reported questionnaire. Finally, a total of 48 participants met the criteria for inclusion in the study. They were randomly assigned to one of two groups: self-awareness condition (SA), or control condition (CC) (SA:14 female and 11 male with a mean ± SD age of 24.16 ± 0.77 years; CC: 13 female and 10 male with a mean ± SD age of 23.67 ± 0.85 years; *t* = −0.51, *p* > 0.05). There was no significant difference between the two groups with respect to PSS (*t* = −1.23, *p* > 0.05). Additionally, to exclude the possible intellectual differences between the two groups, we assessed participants’ IQ using the Raven’s Standard Progressive Matrices (*t* = 1.24, *p* > 0.05). The study and recruitment of participants were approved by the Ethics Committee of the local university and performed in strict accordance with the approved guidelines.

### Questionnaires

Perceived stress was assessed by Cohens PSS (10-item version) (Cohen, 1988). The scale measures perceived stress over the last month on a 5-point Likert scale ranging from 0 (never) to 4 (always). The exemplary item is: “In the last month, how often have you felt that you were unable to control the important things in your life?” PSS has been frequently used to measure chronic stress (Gianaros et al., 2007; Orem et al., 2008; González-Ramírez et al., 2013).

SLSI (Gadzella, 1994) is a self-reported scale, which was used to assess the stressors (frustration, stress, change, etc) of college students, as well as the physiological, behavioral, cognitive, and emotional reactions of individuals under stress. The exemplary item is: “As a student, I have felt frustration because I did not achieve the goal.” SLSI was controlled to exclude the effect of any other major stressors over the last month (Misra et al., 2000; Misra and Castillo, 2004; Sabih et al., 2013).

### Procedure

After arriving at the laboratory, participants were informed of the experimental procedure and completed the demographic information collection. After application of the electrodes, participants were seated in a dimly lit, sound-attenuated, electrically shielded chamber with a CRT monitor set approximately 60 cm away from participants’ eyes. Participants completed the SST and n-back task while behavioral and EEG data were recorded.

**Figure 1:**
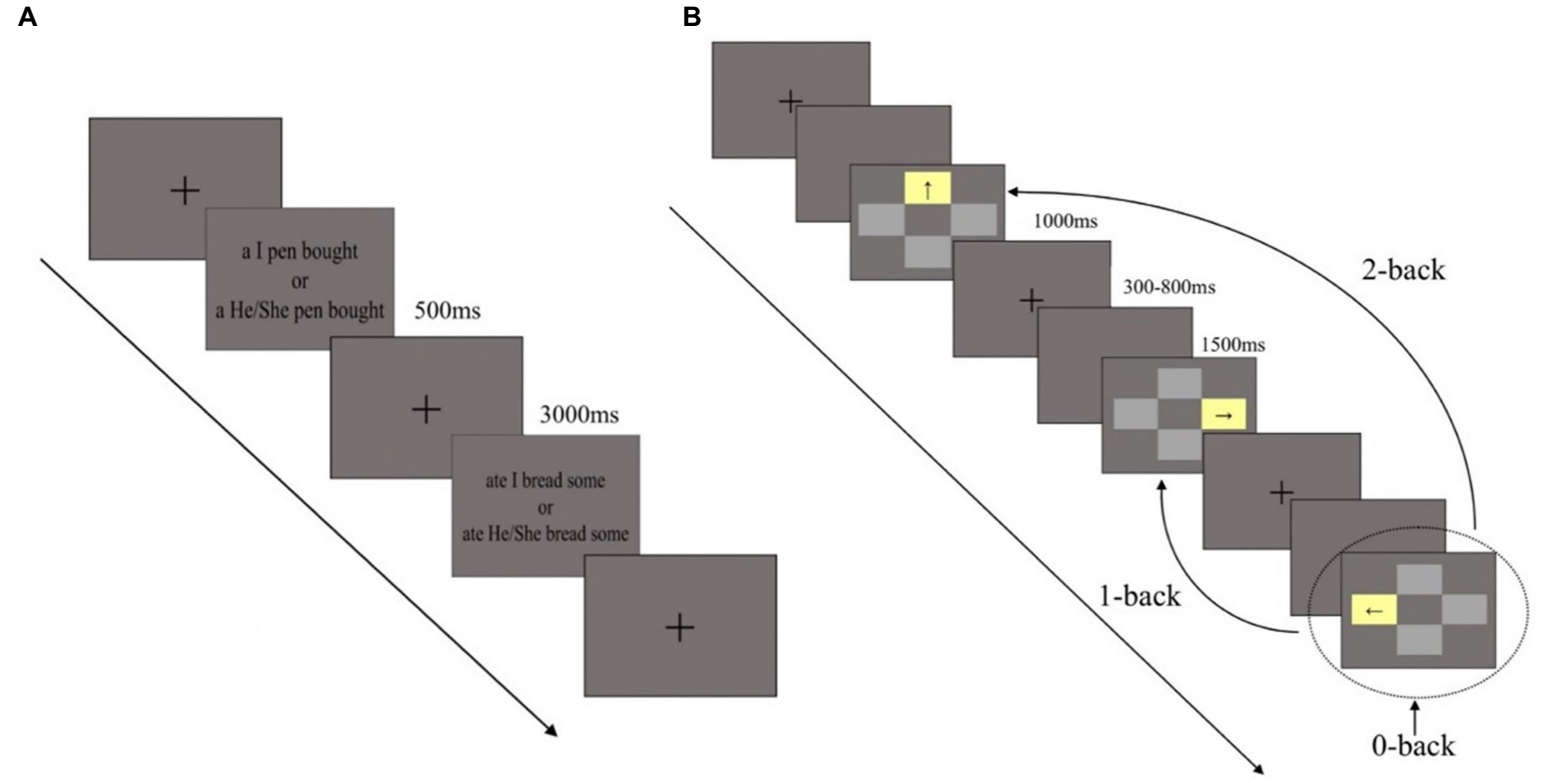
Experimental procedure and design. **(A)** The experimental procedure of priming self-awareness task. **(B)** The experimental procedure of working memory task (0-, 1-, and 2-back). Dashed circle represents the current trial, the curves indicate correct response trials in each load task.

Participants were first asked to finish the SST to increase self-awareness (Xu et al., 2021). In the SST task, participants in the self-awareness group were asked to unscramble 80 scrambled sentences (18° of visual angle) that started with–“I” (e.g., I buy some bread), whereas participants in the control group received 80 scrambled sentences (18° of visual angle) which started with “he” or “she” (e.g., He buys some bread). The only difference between the groups was the use of “I” or “he” or “she.” The task consisted of four blocks, and each block contained 20 trials, resulting in a total of 80 trials. The trials were presented randomly. In each trial, the sentence was presented on the screen for 3,000 ms, and participants answered out loud (Figure 1A).

After the SST task were finished, a spatial n-back task was immediately used to test WM (Chen et al., 2019). Task difficulty varied across three task loads (0-back, 1-back, and 2-back). In the task, four gray rectangles (5° × 3.5° of visual angle) were located above, below, to the left, and to the right of a central fixation cross. One of the gray rectangles would turn yellow in each trial and participants responded to the location of the yellow rectangle in the 0-back load, location of the previous yellow rectangle in the 1-back load, and the location of the yellow rectangle two trials before in the 2-back load. Before the experiment, participants took part in a practice session and received feedback. The experiment did not begin until the participants reached an accuracy rate above 85% at each task load. During the formal experiment, all participants took part in the three tasks (0-, 1-, and 2-back) sequentially without feedback. Participants responded by pressing the corresponding arrow key on the digital keypad (e.g., 8 for above, 5 for below, 4 for left, and 6 for right). In each trial, one of the four gray rectangles turned yellow for at most 1,500 ms if no responses were made, with an inter-stimulus interval of 1,300–1800 ms which was randomly applied. There were three task loads and eight blocks per load. In total, the formal experiment included 24 blocks and each block contained 20 trials. Trials inside each block were presented randomly and the entire task lasted about 15–20 min (Figure 1B). E-Prime software (Version 2.0, Psychology Software Tools, Inc., Pittsburgh, PA, United States) was used to present the stimuli and record the behavioral data.

### Behavioral data analysis

The reaction times (RT) and accuracy (ACC) data for the spatial n-back task were included only when the WM probe was correctly recognized. Removal of these trials resulted in the elimination of 6% of all trials. A repeated-measure analysis of variance (ANOVA) was performed with two (group: self-awareness vs. control) between-subject factors, and three (load: 0-, 1-, and 2-back) within-subject factors.

### Electrophysiological recording and analysis

Brain electrophysiological activity was continuously recorded from a 64-Channel EEG recording system (Brain Products, GmbH, Germany) with references on the middle at FCz. The inter-electrode impedance was always controlled below 10 kΩ. EEG and EOG were amplified using a 0.05–100 Hz band pass filter and continuously sampled at 500 Hz for offline analysis.

Raw EEG data was processed offline using Brain Vision Analyzer version 2.1 (Brain Products, GmbH; Gilching, Germany). For the data analysis, (I) the former reference FCz was reinstated as an additional data channel (Zendel and Alain, 2014); (II) the data was offline re-referenced to the average of all the electrodes; (III) digital filtering with a 30 Hz low-pass and a 0.1 Hz high-pass filtered with a 24-bit analog-to-digital converter; (IV) ocular artifacts were corrected using Independent Component Analysis (ICA) of the continuous data (Makeig et al., 1997); (V) thereafter, ERPs evoked by the rectangles during the study phase were then segmented into 1,200 ms epochs, which began 200 ms before and ended 1,000 ms after stimulus onset; (VI) baseline-corrected with respect to 200 ms pre-stimulus; (VII) trials exceeding ±80 μV were excluded from averaging; (VII) EEGs recorded in the three blocks were averaged separately for each participant, and only trials with correct responses were included in ERP averages. Consequently, the percentages of trials retained in the three task loads were 98.13, 94.34, and 80.96%. Respectively, there was no significant difference between the self-awareness group and control group (0-back: *t* = −1.41, *p* = 0.29; 1-back: *t* = −0.06, *p* = 0.96; 2-back: *t* = 0.48, *p* = 0.68).

Based on previous studies of n-back tasks, P2, N2, and LPP were measured (Lenartowicz et al., 2010; Qi et al., 2016, 2018; Ma et al., 2019). The following five electrode points were selected for statistical analysis: Fz and FCz were chosen for P2 (150–200 ms) (Yuan et al., 2016; Nikolin et al., 2020) and N2 (215–265 ms) (Luck and Hillyard, 1994; Chen and Mitra, 2009), and FCz, Cz and CPz were chosen for LPP (400–700 ms) (Ahonen et al., 2016; Grissmann et al., 2017; Ma et al., 2019). The mean amplitudes of these components were averaged from these electrode sites and then analyzed by repeated-measures ANOVA. Analysis was performed with two (group: self-awareness vs. control) between-subject factors, and three (load: 0-, 1-, and 2-back) within-subject factors. The Greenhouse–Geisser correction was used when the data violated the assumption of sphericity. All statistical analyses of behavioral and ERP data were conducted using SPSS 25.0 (IBM, SPSS Statistics for Windows, Version 25.0. Armonk, NY: IBM Corp.).

In order to explore whether self-awareness has a protective effect on WM in people under chronic stress, and the neural mechanism involved, correlational analyses involving associations between the PSS, behavioral performance measures (i.e., ACC and RT), and P2/N2/LPP were conducted. Moreover, the mediation model was tested to examine whether neural processing (P2/N2/LPP) mediated the expected relationships between self-awareness and behavior. In the model, the groups (SA/CC) were entered as the independent variable, P2, N2 and LPP as the mediators respectively, and the dependent variable was the behavioral performance (i.e., ACC and RT) of the 0-, 1-and 2-back tasks, respectively. The analyses were performed using the SPSS macro from Preacher and Hayes (2008). In accordance with the recommendations outlined in Preacher and Hayes (2004), a bootstrapping procedure involving 5,000 samples with a 0.95 confidence interval (CI) was used to test for indirect effects (Preacher and Hayes, 2004, 2008).

## Results

### Behavioral results

**Figure 2:**
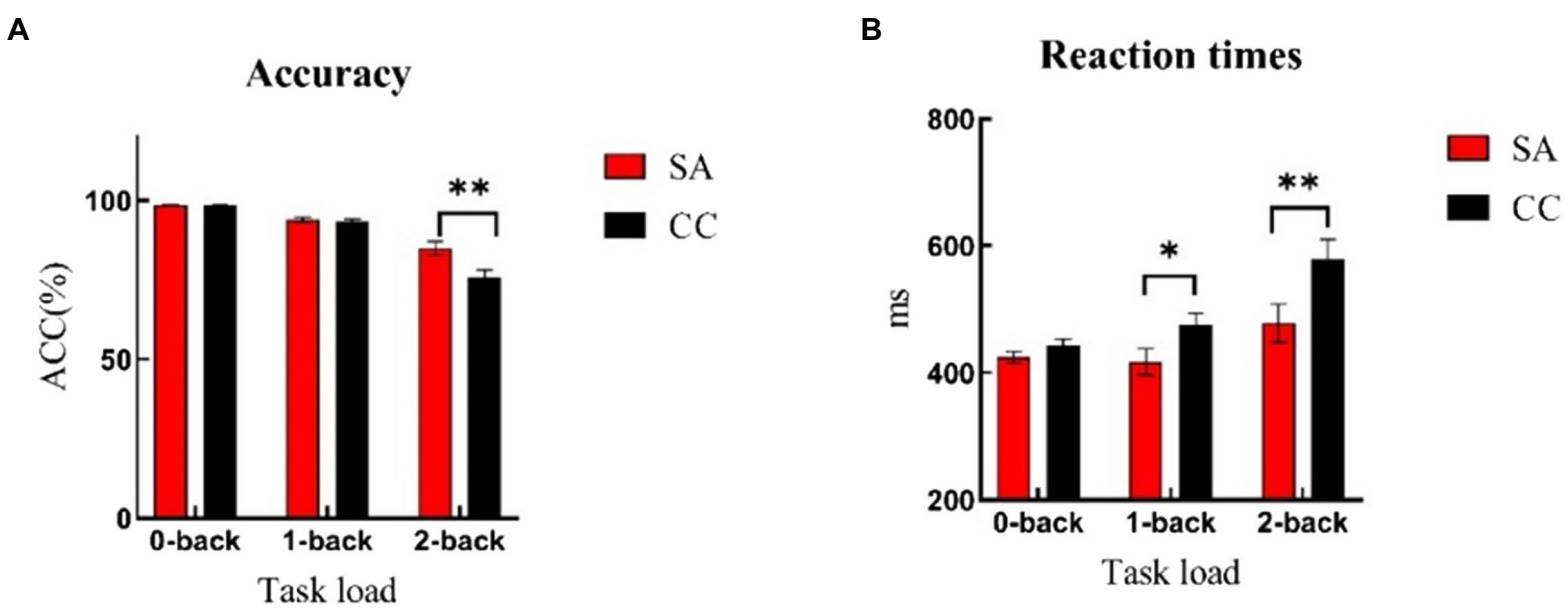
Accuracy and reaction times of n-back task under the self-awareness and control groups. Error bar represents the standard error. **(A)** Self-awareness group showed better accuracy compared with controls in 2-back task. **(B)** Control group reacted faster than controls both in 1-back and 2-back tasks. *p < 0.05; **p < 0.01. Red rectangle represents participants of self-awareness group (SA), and black rectangle represents participants of control group (CC).

The ACC showed a significant main effect of load (0-, 1-, and 2-back) [*F*(2,92) = 102.84, *p* < 0.01, η^2^ = 0.69]. *Post hoc* analysis showed ACC of 0-back (SA: *M* = 98.46, *SD* = 0.41; CC: *M* = 98.49, *SD* = 0.36) was significantly higher than ACC of 1-back (SA: *M* = 94.11, *SD* = 3.21; CC: *M* = 93.46, *SD* = 3.47; *p* < 0.01) and 2-back (SA: *M* = 85.08, *SD* = 10.31; CC: *M* = 75.78, *SD* = 12.19; *p* < 0.01). ACC of 1-back was significantly higher than 2-back, *p* < 0.01. There was a main effect of group, [*F*(1,46) = 6.91, *p* = 0.01, η^2^ = 0.13], showing higher accuracy in SA compared with CC. The interaction of load and group was also significant [*F*(2,92) = 7.92, *p* < 0.01, η^2^ = 0.15]. Simple effect analysis showed at 2-back load, the ACC of self-awareness group was significantly higher than those of control group [*F*(1,46) = 8.13, *p* < 0.01], while there were no significant differences at 0-back [*F*(1,46) = 0.05, *p* = 0.81] and 1-back [*F*(1,46) = 0.46, *p* = 0.50] (see Figure 2A; Table 1-ACC).

**Table 1:** Descriptive statistics of accuracy and response time (M ± SD).

0 – back			1 – back		2 – back	
	ACC (100%)	RT (ms)	ACC (100%)	RT (ms)	ACC (100%)	RT (ms)
Self-awareness	98.46 ± 0.41	424.59 ± 42.71	94.11 ± 3.21	417.13 ± 102.02*	85.08 ± 10.31**	478.38 ± 147.49**
Control	98.49 ± 0.36	443.09 ± 52.85	93.46 ± 3.47	474.02 ± 100.81*	75.78 ± 12.19**	578.35 ± 158.90**

ACC stands for accuracy and RT stands for reaction time. ACC significant in 2-back. RT significant in 1-back and 2-back. *p < 0.05; **p < 0.01.

For response times, the main effect of load was significant [*F*(2,92) = 16.92, *p* < 0.01, η^2^ = 0.27]. *Post hoc* analysis revealed that RT of 2-back (SA: *M* = 478.38, *SD* = 147.49; CC: *M* = 578.35, *SD* = 158.90) was significantly longer than RT of 0-back (SA: *M* = 424.59, *SD* = 42.71; CC: *M* = 443.09, *SD* = 52.85; *p* < 0.01) and 1-back (SA: *M* = 417.13, *SD* = 102.02; CC: *M* = 474.02, *SD* = 100.81; *p* < 0.01). There was no difference between 0-back and 1-back, *p =* 0.33. The main effect of group was significant [*F*(1,46) = 5.85, *p* = 0.02, η2 = 0.11], showing shorter response times in SA participants compared with controls. The load × group interaction effect was marginally significant [*F*(2,92) = 2.65, *p* = 0.07, η^2^ = 0.06]. Simple effect analysis showed that in comparison with 0-back task [*F*(1,46) = 1.78, *p* = 0.19], the response time of self-awareness group was significantly faster than control group at 1-back [*F*(1,46) = 3.78, *p* = 0.05] and 2-back [*F*(1,46) = 5.10, *p* = 0.03] loads (see Figure 2B; Table 1-RT).

### ERP results

#### P2

**Figure 3:**
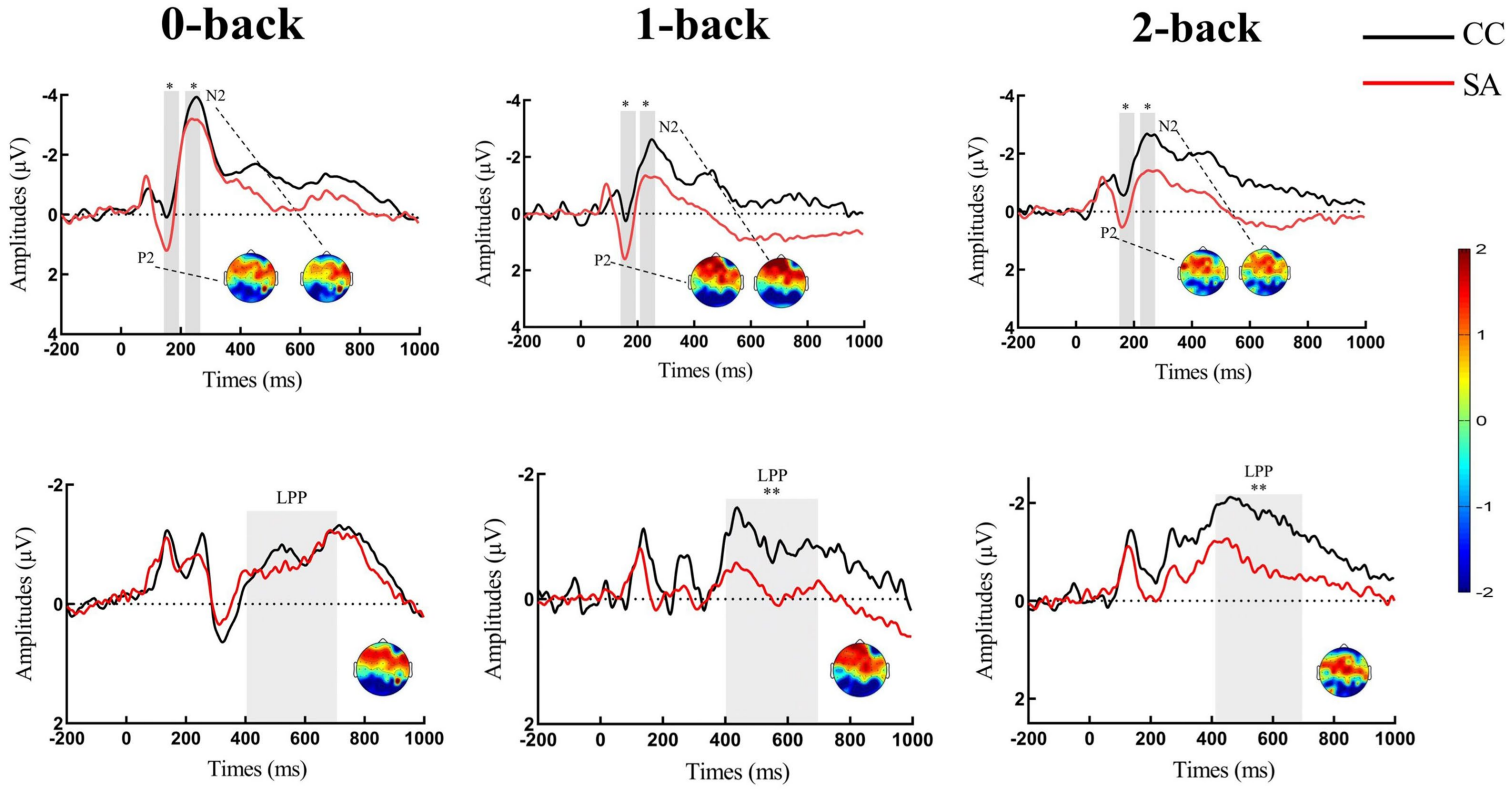
Grand-averaged event-related potential waveforms for P2/N2 and LPP of n-back task in self-awareness and control groups. Fz and FCz were selected for P2 (shaded: 150–200 ms time window) and N2 (shaded: 215–265 ms time window). FCz/Cz and CPz were selected for LPP (shaded: 400–700 ms time window). Scalp topographies of n-back task in self-awareness and control groups were come from difference waveforms (SA-CC) which were selected from a time window of 150–200 ms for P2, 215–265 ms for N2, and 400–700 ms for LPP. Please note that the positive-going component is plotted on the lower side of the y-axis, while the negative-going component is plotted on the upper side of the y-axis. *p < 0.05; **p < 0.01. Red line represents participants of self-awareness group (SA), and black line represents participants of control group (CC).

**Figure 4:**
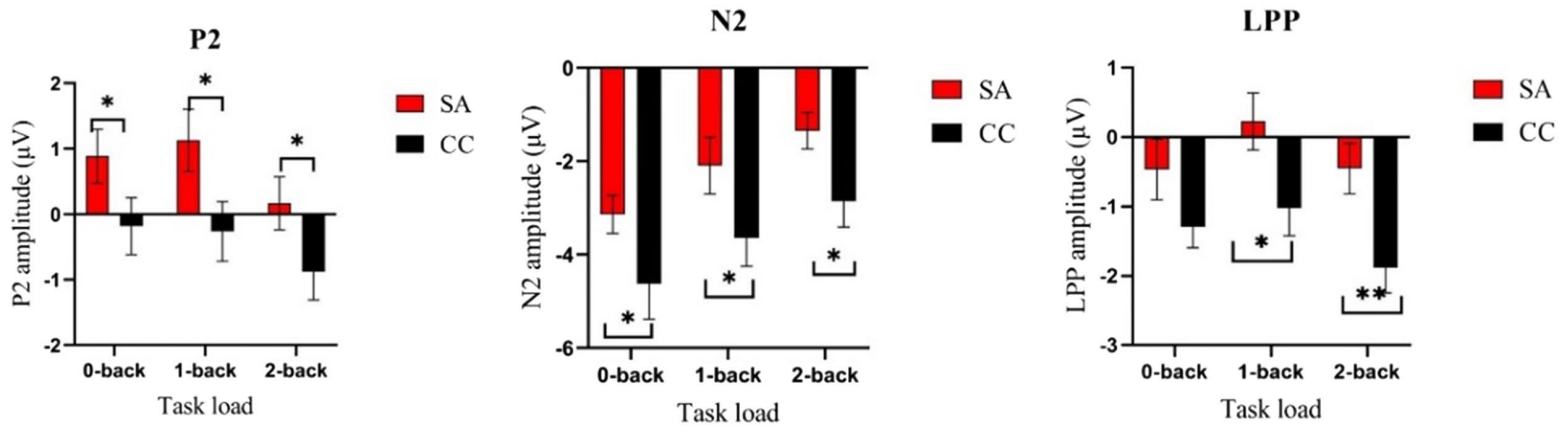
Comparison of amplitudes of P2, N2, and LPP in n-back task under self-awareness and control groups. Error bar represents the standard error. *p < 0.05; **p < 0.01. The self-awareness group evokes larger P2/LPP than the control group. The control group evokes larger N2 than the self-awareness group. Red rectangle represents participants of self-awareness group (SA), and black rectangle represents participants of control group (CC).

Repeated-measures ANOVA revealed a significant main effect of load for P2 amplitude [*F*(2,92) = 14.98, *p* < 0.01, η^2^ = 0.25]. *Post hoc* analysis showed that 2-back (SA: *M* = 0.17, *SD* = 1.98; CC: *M* = −0.87, *SD* = 2.11) evoked smaller P2 than 0-back (SA: *M* = 0.89, *SD* = 2.01; CC: *M* = −0.18, *SD* = 2.12; *p* < 0.01) and 1-back (SA: *M* = 1.13, *SD* = 2.33; CC: *M* = −0.26, *SD* = 2.23; *p* < 0.01). There was no difference between 0-back and 1-back, *p =* 0.66. The main effect of group was significant [*F*(1,46) = 3.94, *p* = 0.05, η^2^ = 0.08], showing increased P2 in SA participants compared with controls. The group × load interaction was not significant [*F*(2,92) = 0.74, *p* = 0.47] (see Table 2; Figures 3, 4).

**Table 2:** Descriptive statistics of amplitudes of P2/N2/LPP (M ± SD).

0 – back				1 – back			2 – back		
	P2 (μV)	N2 (μV)	N2 (μV)	P2 (μV)	N2 (μV)	LPP (μV)	P2 (μV)	N2 (μV)	LPP (μV)
Self-awareness	0.89±2.01*	−3.13±2.00*	−0.47±2.14	1.13±2.33*	−2.09±2.96*	0.22±2.02*	0.17±1.98*	−1.34±1.89*	−0.45±1.77**
Control	−0.18±2.12*	−4.62±3.72*	−1.29±1.47	−0.26±2.23*	−3.64±2.97*	−1.02±1.98*	−0.87±2.11*	−2.86±2.69*	−1.88±1.79**

The data is the grand-average ERP waveforms. P2 (Fz/FCz), N2 (Fz/FCz), and LPP (FCz/Cz/CPz). The reported electrodes provided here are in accordance with those in Figures 3, 4. *p < 0.05; **p < 0.01.

#### N2

The amplitude on the frontal N2 component revealed a significant main effect of load [*F* (2,92) = 25.64, *p* < 0.01, η^2^ = 0.36]. *Post hoc* analysis showed that 0-back (SA: *M* = −3.13, *SD* = 2.00; CC: *M* = −4.62, *SD* = 3.72) evoked larger N2 than 1-back (SA: *M* = −2.09, *SD* = 2.96; CC: *M* = −3.64, *SD* = 2.97; *p* < 0.01) and 2-back (SA: *M* = −1.34, *SD* = 1.89; CC: *M* = −2.86, *SD* = 2.69; *p* < 0.01). The amplitude of 1-back was significantly larger than 2-back, *p* < 0. 01. The main effect of group was significant (*F* (1, 46) = 4.11, *p* = 0.04, η^2^ = 0.08), showing a smaller N2 amplitude in SA than it in CC. The group × load interaction was not significant [*F*(2,92) = 0.01, *p* = 0.99] (see Table 2; Figures 3, 4).

#### LPP

Analysis of LPP amplitudes revealed that the main effect of load [*F*(2,92) = 4.63, *p* = 0.01, η^2^ = 0.09] was significant. *Post hoc* analysis revealed that 1-back (SA: *M* = 0.22, *SD* = 2.02; CC: *M* = −1.02, *SD* = 1.98) evoked more positive LPP than 2-back (SA: *M* = −0.45, *SD* = 1.77; CC: *M* = −1.88, *SD* = 1.79), *p* < 0.01. There was no difference between 0-back (SA: *M* = −0.47, *SD* = 2.14; CC: *M* = −1.29, *SD* = 1.47) and 1-back, *p =* 0.10, as well as 0-back and 2-back, *p =* 0.31. For the main effect of group [*F*(1,46) = 6.63, *p* = 0.01, η^2^ = 0.13], we observed significantly increased LPP amplitudes in SA individuals than in controls. The group × load interaction was also not significant [*F*(2,92) = 2.39, *p* = 0.11] (see Table 2; Figures 3, 4).

### Correlations analysis results

**Figure 5:**
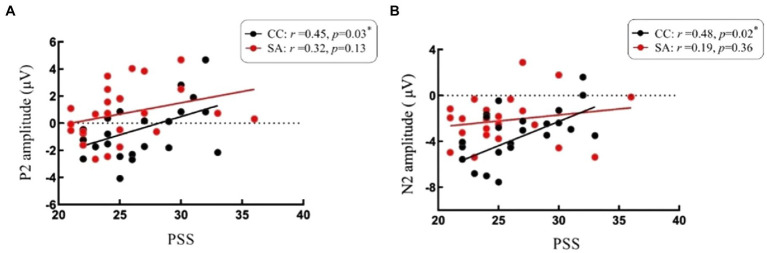
Scatterplots depicting correlations between self-perceived stress level and ERP responses. **(A)** Correlations between P2 and scores of Perceived Stress Scale (PSS). **(B)** Correlations between N2 and scores of Perceived Stress Scale (PSS). *p < 0.05. Red spot and line represent participants of self-awareness group (SA). Black spot and line represent participants of control group (CC).

We found a significant positive relationship between self-perceived stress and P2 in CC, and higher PSS scores were associated with larger P2 (*r* = 0.45, *p* = 0.03). However, there was no significance in SA (*r* = 0.32, *p* = 0.13). Follow-up analysis revealed the same positive relationship found in N2. The N2 under CC was significantly positively correlated with PSS (*r* = 0.48, *p* = 0.02), while not significantly associated with PSS in SA (*r* = 0.19, *p* = 0.36). See Figure 5 for scatter plots of the reported correlations.

### Mediation analysis results

**Figure 6:**
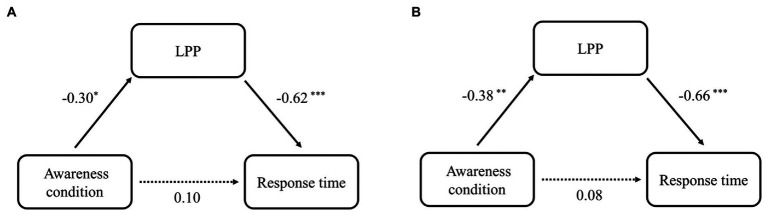
The mediating effect of LPP in the influence of awareness group (self-awareness and control) on RT at 1-back **(A)** and 2-back **(B)**. The values indicate standardization coefficients (Beta). *p < 0.05; **p < 0.01; ***p < 0.001.

The mediation results showed a complete mediating effect of LPP on the association between awareness group and RT at 1-back and 2-back loads, respectively. Specifically, for 1-back load, awareness group (Self-Awareness and Control) significantly predicted LPP (β = −0.30, *SD* = 0.58, *t* [46] = −2.16, *p* = 0.04). 95% confidence intervals (CI) were [−2.40, −0.08]. Meanwhile, LPP also significantly predicted the RT at 1-back (β = −0.62, *SD* = 5.84, *t* [46] = −5.33, *p* < 0.01). 95% CI was [−42.03, −17.24]. The indirect effect of awareness group on RT was significant, 95% CI was [4.81, 68.77], while the zero was included in 95% CI of direct effect [−30.81, 70.84]. For 2-back load, awareness group (Self-Awareness and Control) significantly predicted LPP (β = −0.38, *SD* = 0.51, *t* [46] = 2.55, *p* < 0.01). 95% confidence intervals (CI) were [−2.46, −0.39]. Furthermore, LPP also significantly predicted the RT (β = −0.66, *SD* = 9.28, *t* [46] = −6.02, *p* < 0.01) loads. 95% CI was [−73.72, −33.08]. The indirect effect of awareness group on RT was also significant, 95% CI was [24.61, 137.86]. The zero was included in 95% CI of direct effect [−52.57, 100.33], which was not significant. See Figure 6 for a visual depiction of the models with effect sizes of the direct and indirect paths.

## Discussion

We investigated whether self-awareness protects WM in people under chronic stress and the neurocognitive processes involved by using the n-back task. The results of the present study confirm the presence of some behavioral and electrophysiological differences. The main finding was that after self-awareness priming, WM performances of individuals under chronic stress were enhanced, as measured by task accuracy and response times. EEG analyses revealed that P2 in self-awareness participants was more positive than that in control participants during all n-back loads, while N2 decreased. Meanwhile, LPP was significantly larger during 1-and 2-back tasks. Critically, LPP mediated the negative correlation between self-awareness inducement and WM response times.

The present study found that participants of the self-awareness group reacted faster and more accurately in the n-back task, suggesting that a priming of self-awareness is conducive to the performance of WM tasks. According to the motivational intensity theory, attentional resources are mobilized proportional to the cognitive task difficulty as long as success is important and worthwhile (Brehm and Self, 1989; Wright and Kirby, 2001). On the other hand, priming self-awareness would heighten sensitivity to social standards, which virtually increases the importance of success. Silvia and Phillips (2013) found that self-awareness participants had a higher accuracy or faster response times in cognitive tasks. Their finding was in line with other studies, suggesting that self-awareness significantly enhanced motivation or self-regulation and resulted in better performance with different task demands (Liebling and Shaver, 1973; Alberts et al., 2011; Huang et al., 2017; Bender et al., 2018).

For the ERP data, we observed dissociable effects at the early encoding stage, showing that there was larger P2 and smaller N2 in self-awareness participants than in controls. The results are consistent with our hypothesis. P2 is thought to be related to the early allocation of attention and initial stage of context updating (Thorpe et al., 1996; Lefebvre et al., 2005, 2010). P2 has been reported in several types of WM tasks, with larger P2 always being associated with efficient attention selection (Lenartowicz et al., 2014; Yuan et al., 2016; Vila-Ballo et al., 2018). Qi et al. (2016) observed larger P2 in the control group than in the stress group (Qi et al., 2016). Along similar lines, Li et al. (2020) revealed that participants who reacted faster and more accurately evoked larger P2 (Li et al., 2020). In the present study, the increased P2 in the self-awareness group relative to control group may reflect that self-awareness enhanced the allocation of attention resources at the encoding stage. It is likely that more attention resources were allocated to target stimuli when self-awareness was induced.

N2 is the index related to the attention control process (Luck and Hillyard, 1994; Kreusch et al., 2014; Couperus et al., 2021). The N2 enhancement can be interpreted as reflecting attention investment in target and non-target discrimination processing (Näätänen and Picton, 1986). A similar interpretation was proposed by Sänger Bechtold et al. (2014) who argued that a larger N2 may therefore be indicative of more attention resources being required for discrimination and selection during cognitive tasks. Our results, to some extent, supported this idea. The enhanced N2 in the control group might suggest that a more intensive attention control process was triggered under the chronic stress state, in which more attention resources must be allotted to complete encoding tasks adequately. Combining the P2 and N2 output, self-awareness might push individuals to allocate more attention resources to target stimuli instead of non-target information in the encoding stage. Such an interpretation was also supported by our behavioral results.

Late positive potential differed significantly between the groups, as expected. LPP in the self-awareness group was significantly larger than that in the control group. LPP is a dynamic measure of the sustained allocation of attentional resources to visual stimuli at the maintenance stage of WM (Ruchkin et al., 1990; Hajcak et al., 2010; Weinberg and Hajcak, 2010). Some studies have argued that decreased LPP is a sign of the difficulty of maintaining information in a n-back task or target detection task when there are simultaneous influxes of a variety of information (Macnamara et al., 2011; Ma et al., 2019). On the other hand, according to attentional control theory, stress restrains the allocation of attentional resources toward task-relevant stimuli (Bishop et al., 2004; Eysenck et al., 2007; Bishop, 2008). However, participants in the self-awareness state recovered their concentration of attention and allocated more attentional resources to the task-relevant stimuli.

Additionally, we found evidence in the results of the correlations. We found a highly positive correlation between P2/N2 and the PSS scores for individuals under chronic stress (i.e., CC), indicating that individuals who have higher stress perception will evoke larger P2/N2, the self-perceived stress plays a critical role in the modulation of P2/N2 components for individuals who are under chronic stress. Those results are supported by a prior n-back task study (Yuan et al., 2016). However, in our study, when inducing self-awareness (i.e., SA), such a key role disappeared. On the other hand, P2 and N2 are associated with the early encoding stage (Li et al., 2020; Peng et al., 2020). Therefore, we speculated that the protective effect of self-awareness on WM at the encoding stage may be *via* reduction of the sensitivity of stress. Of course, this speculation requires further research.

The mediation analyses revealed that LPP mediated the relationships between the self-awareness-group and the RT of both 1-and 2-back tasks. LPP has been linked to memory maintenance, reinterpretation of stimulus meaning, as well as attentional biases as measured by RT (Koenig and Mecklinger, 2008; MacNamara et al., 2009; Gable and Harmon-Jones, 2010). Evidence from a series of WM tasks suggests that WM load has negative effects on concentration and the maintenance of attention, as measured by LPP (Weinberg and Greg, 2011). Specifically, LPP was larger on low-load than on high-load trials with a shorter RT (Flaisch et al., 2008a, 2008b). Taken together, it was speculated that the RT to targets would be specifically reflected in the magnitude of the LPP. In the present study, compared with the control group, larger LPP in the self-awareness group may reflect more attention resources investment in ongoing maintenance processes, possibly underlying the nature of the protective effects of self-awareness on WM process.

The key contribution of this work is that it is the first attempt to combine self-awareness, WM, and chronic stress and provides a new idea for the protection of WM capacity in individuals under chronic stress. We verified that self-awareness indeed endows individuals with higher WM performances. Furthermore, with the advantage of high temporal resolution in ERPs, we explored the protective mechanism of self-awareness on WM capacity at the neural dynamics level. Exploring the protective role of self-awareness in WM processes in people under chronic stress is helpful to have a deeper understanding of individuals under chronic stress, so as to develop specific training and treatment programs.

## Limitations and future directions

There are some limitations to the current study. First, our data were taken only from the results of one single intervention; however, the effects of chronic stress are long-term. Our modulation pathway would follow from longitudinal data and improve the level of trait self-awareness through a longer-term training. Second, we used scrambled sentences for priming self-awareness in the current study. In future research, a variety of paradigms and techniques for priming self-awareness should be developed. Other techniques, such as simultaneous EEG-fMRI and MEG, can be used, which would enable us to more directly examine the interplay of the PFC, self-awareness and WM tasks in people under chronic stress. Lastly, although the present work provides a first look at the protective effect of self-awareness for WM capacity, future research with larger and more representative participant samples are needed to establish a better understanding of these effects.

## Conclusion

We found that self-awareness protects WM from the adverse effects of chronic stress, which was accomplished by enhancing allocation and mobilization of attention. We found significantly better performance in self-awareness group participants, measured by accuracy and reaction times. The increase in P2, decrease in N2, and increase in LPP were consistent with our hypothesis. These findings suggest that the protective effect of self-awareness may occur at the encoding and maintenance stages in the WM process. Mediational analyses showed that the LPP mediated the relationship between awareness condition and WM performance (i.e., RT). This result potentially suggests that the neural mechanism of the protective effect of self-awareness on WM in people under chronic stress was exerted by improving maintenance processing.

## Data availability statement

The raw data supporting the conclusions of this article will be made available by the authors, without undue reservation.

## Ethics statement

The studies involving human participants were reviewed and approved by Ethics Committee of Qilu Normal University. The patients/participants provided their written informed consent to participate in this study.

## Author contributions

ZW and YL had substantial contributions to the conception and design of the work. SZ and SH had substantial contributions to the acquisition, analysis, and interpretation of data for the work. DJ, SH, and YL had revising it critically for important intellectual content. WX participated in the whole process. All authors contributed to the article and approved the submitted version.

## Funding

This work was supported by NSFC (31920103009), the Major Project of National Social Science Foundation (20&ZD153), and Shenzhen-Hong Kong Institute of Brain Science -Shenzhen Fundamental Research Institutions (2022SHIBS0003).

## Conflict of interest

The authors declare that the research was conducted in the absence of any commercial or financial relationships that could be construed as a potential conflict of interest.

## Publisher’s note

All claims expressed in this article are solely those of the authors and do not necessarily represent those of their affiliated organizations, or those of the publisher, the editors and the reviewers. Any product that may be evaluated in this article, or claim that may be made by its manufacturer, is not guaranteed or endorsed by the publisher.
